# Mapping paths: new approaches to dissect eukaryotic signaling circuitry

**DOI:** 10.12688/f1000research.8818.1

**Published:** 2016-07-27

**Authors:** Nebibe Mutlu, Anuj Kumar

**Affiliations:** 1 Department of Molecular, Cellular and Developmental Biology, University of Michigan, Ann Arbor, MI, USA

**Keywords:** signaling pathways, eukaryotic signaling networks, CRISPR/Cas, proteomics, protein interactions, cell signaling, yeast

## Abstract

Eukaryotic cells are precisely “wired” to coordinate changes in external and intracellular signals with corresponding adjustments in the output of complex and often interconnected signaling pathways. These pathways are critical in understanding cellular growth and function, and several experimental trends are emerging with applicability toward more fully describing the composition and topology of eukaryotic signaling networks. In particular, recent studies have implemented CRISPR/Cas-based screens in mouse and human cell lines for genes involved in various cell growth and disease phenotypes. Proteomic methods using mass spectrometry have enabled quantitative and dynamic profiling of protein interactions, revealing previously undiscovered complexes and allele-specific protein interactions. Methods for the single-cell study of protein localization and gene expression have been integrated with computational analyses to provide insight into cell signaling in yeast and metazoans. In this review, we present an overview of exemplary studies using the above approaches, relevant for the analysis of cell signaling and indeed, more broadly, for many modern biological applications.

Wild-type cell growth and function require the precise processing of both intrinsic and external signals, resulting in the regulation of nucleic acid/protein networks that coordinate genetic, physiological, and biochemical changes necessary for cellular adaptation. Mutations in these signaling networks are the hallmark of numerous cancers and diseases; consequently, such signaling pathways have been the focus of extensive research interest ^1– 3^. Signaling circuits have been studied for many decades through methods that are now classic, encompassing the application of forward and reverse genetic screens as well as targeted studies to identify gene-to-gene relationships regulating transcription, translation, and post-translational modifications. These methods have provided a wealth of data, but, with so much regarding these signaling circuits still to be uncovered, the need persists for improved methods to more fully dissect signaling pathways. In this review, we highlight some of the most promising recent trends in the analysis of cell signaling circuits and discuss selected recent studies exemplifying these trends. In particular, we review ongoing advances for precise chromosomal DNA manipulation in higher eukaryotes and improved methods for the quantitative analysis of the proteome and its dynamics. We additionally present an overview of studies integrating multiple laboratory and bioinformatic analyses and a focus on the analysis of signaling at the level of single cells.

## Genetic screening with the CRISPR/Cas system

**Figure 1.**
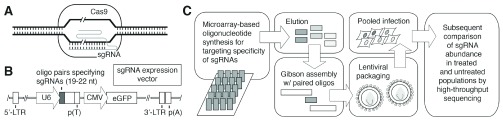
An overview of CRISPR/Cas-based screening. ( **A**) A simplified representation of target DNA cleavage by single-guide RNA (sgRNA)-directed Cas9 nuclease activity. Double-stranded DNA cleavage is indicated by the slash marks. ( **B**) A typical vector for expression of the sgRNAs is presented. The U6 Pol III promoter for expression of the sgRNA is indicated with an arrow labeled “U6” to the left of the diagram. Adapted from Zhou *et al*. ^57^. ( **C**) Overview of an approach for sgRNA library construction. Oligonucleotides corresponding to the region of the sgRNA providing target specificity may be synthesized commercially on microarrays. Oligonucleotides are eluted from the microarray, and Gibson assembly-based cloning with the eluted oligonucleotides and overlapping nucleic acid fragments can be used to construct the library of sgRNA expression constructs for subsequent lentiviral packaging. Target cells are transduced with the lentiviral sgRNA pools to generate mutants for screening as indicated. Abbreviations: CMV, cytomegalovirus; CRISPR/Cas, clustered regularly interspaced short palindromic repeats/CRISPR-associated; eGFP, enhanced green fluorescent protein; LTR, long terminal repeat; nt, nucleotide.

In dissecting cell signaling responses, a critical first step often entails identifying constituent genes encoding products required for the signaling event. Traditionally, gene functions have been identified broadly by using forward genetic screens, wherein phenotype-to-genotype relationships are established through the generation of non-specific mutations and subsequent selection/screening of mutants exhibiting a desired phenotype ^4^. Reverse genetic screens have become much more commonly employed over the last 25 years, particularly for screens in organisms amenable to facile genetic manipulation. The budding yeast *Saccharomyces cerevisiae* stands as an excellent example. In yeast, polymerase chain reaction-based approaches have been used to generate DNA with a selectable marker flanked by a sequence that can be used to target the DNA to a precise locus upon chromosomal integration by homologous recombination; the resulting mutant allele, often a gene deletion, can be screened for any number of desired phenotypes in stable haploid or diploid yeast ^5, 6^. Extensive libraries of mutant alleles have been constructed for reverse genetics in the budding yeast by this method as well as through methods encompassing transposon mutagenesis ^7– 10^. Transposon mutagenesis has also been used to generate mutant allele collections in metazoans ^11– 13^, although more recent reverse genetic screens in higher eukaryotes have predominantly used RNA interference-based approaches to reduce expression of target genes ^14, 15^, and additional notable studies have used zinc-finger nucleases and transcription activator-like effector nucleases (TALENs) for the generation of mutant alleles ^16– 18^. The recent discovery and application of CRISPR/Cas-based systems, however, has provided researchers with arguably the most promising tool to date for the manipulation of metazoan genes with ease and specificity ^19, 20^. For thorough reviews of the basics of genome editing via CRISPR/Cas, see the indicated articles ^21, 22^. Figure 1 presents an overview of typical steps in generating a library of single-guide RNAs (sgRNAs) and its application for CRISPR/Cas screening. Recently, CRISPR/Cas-based methods have been employed with great success for genome editing and phenotypic screening in a wide variety of organisms, including mice, flies, zebrafish, and human cells ^23– 30^. Below, we review a few exceptional studies using CRISPR/Cas approaches for phenotypic analysis in mouse and human cell lines.

In a recent landmark study, Chen *et al*. ^31^ used CRISPR/Cas in mice to detect loss-of-function mutations that contribute to tumor growth and lung metastasis. In this work, the researchers constructed a pooled genome-wide library of 67,405 sgRNAs, and this lentiviral library was transduced into a cell line capable of inducing tumors, but not typically metastasis, upon transplantation into mice; this cell line was also transduced with Cas9-enhanced green fluorescent protein (Cas9-eGFP). The pool of mutant cells resulting from this transduction was transplanted into mice, and subsequent analyses using deep sequencing identified sgRNAs enriched in the late-stage primary tumor and lung metastases. Thus, the screen enabled the identification of mutants enriched in metastasis induced by the respective sgRNA. In sum, this study presents several important findings. In particular, analysis of the targets of these sgRNAs revealed novel and known tumor suppressors. Furthermore, characterization of the effects of individual sgRNAs and comparison of primary tumors and metastases offer an explanation for the long-standing question of why the probability of metastases correlates with the size of the primary tumor. Results from this work suggest that the ability to proliferate enhances the ability to metastasize more rapidly. In sum, this work offers a guideline for *in vivo* CRISPR/Cas loss-of-function screens while also validating that animal models generated through this tool provide data relevant to the understanding of human disease.

In another impactful study, Parnas *et al*. ^32^ used a protein marker-based CRISPR screen in mouse primary dendritic cells to determine the regulators of host innate immune response against bacterial lipopolysaccharide (LPS). The immune response against LPS induces an inflammatory cytokine, tumor necrosis factor (TNF). Rather than cell viability, TNF staining was used as a readout in this study for immune response activity after the addition of LPS. By flow cytometry, researchers isolated the aberrantly stained cells resulting from under- or over-induction of the immune response, thereby identifying three clusters of proteins that regulate innate immune response.

Subsequent to early CRISPR/Cas-based loss-of-function screens in human cells ^23, 24^, many groups have implemented similar approaches for functional genomics in human cell culture. Recently, Ma *et al*. ^33^ screened for genes required for cell death induced by West Nile virus by using a library of 77,406 sgRNAs targeting 20,121 genes. Through two rounds of screening, the researchers found that knockout of a subset of seven genes in the endoplasmic reticulum-associated protein degradation (ERAD) pathway protected against virus-induced cell death. Also in 2015, Hart *et al*. used a CRISPR/Cas9 sgRNA library for fitness screens in five human cell lines ^34^, defining fitness phenotypes as a defect in proliferation. For this study, the researchers generated a sgRNA library that targeted, in particular, human protein-coding genes as well as a set of random controls. Using this library coupled with sensitive Bayesian computational methods, Hart *et al*. identified approximately fivefold more fitness genes than had been previously observed.

Although CRISPR/Cas-based systems have emerged as the most popular and exciting tools for genome manipulation in mammals, these systems are still far from perfect. Off-target effects from guide RNAs may confound the interpretation of results ^35^, necessitating appropriate controls. To minimize false-positive results from CRISPR/Cas-derived screens, initial results from a primary screen may be validated through a secondary targeted screen using sgRNAs for only those genes identified in the initial analysis. In total, CRISPR/Cas remains a technology undergoing great development and expansion of application. CRISPR/Cas technologies hold vast potential, both singly as well as in combination with other genome editing tools. For example, CRISPR interference studies, using a catalytically inactive and endonuclease-deficient Cas9 to regulate transcription of target genes in an RNA-guided method, have proven effective in gene silencing studies ^36, 37^.

Interesting alternatives to the CRISPR/Cas9 system are beginning to emerge as well. Notably, Gao *et al*. ^38^ have presented a DNA-guided genome editing system using the *Natronobacterium gregoryi* Argonaute endonuclease for the generation of precise mutations in human cells. *N. gregoryi* Argonaute binds 5’-phosphorylated single-stranded guide DNA oligomers of approximately 24 residues and efficiently generates site-specific double-strand breaks upon loading with the guide DNA. Initial results indicate that the system exhibits a low tolerance to guide-target mismatches as well as efficiency in editing regions with high G/C content. Thus, in the immediate future, the application of newly developed gene editing platforms using CRISPR/Cas-based approaches as well as alternative methods is expected to yield an exciting volume of data deciphering previously uncovered metazoan signaling circuitry.

## Quantitative proteomics

In addition to analyses of signaling pathways at the genetic level, protein-based or proteomic studies (or both) have proven to be highly informative in dissecting eukaryotic signaling pathways. Over the last two decades, the proteomics field has advanced substantially toward determining the protein landscape of the cell at a specific time under specific conditions. Recently, this proteomic “snapshot” has been furthered to provide a quantitative as well as dynamic representation of protein abundance and localization in eukaryotes.

With published reports already presenting a draft catalog of the human proteome ^39– 41^, current research efforts are now more aggressively addressing the dynamics of protein abundance and interactions. Relative to the results from steady-state proteomic studies, data sets presenting protein interaction dynamics may provide unique insight into the signaling events occurring in human cells. In 2015, Huttlin *et al*. ^42^ investigated the human interactome, the proteome-wide set of interactions in a given cell, through affinity purification of a large set of tagged proteins followed by mass spectrometry (termed AP-MS). In this work, the researchers infected 293T cells with a lentiviral library of FLAG-HA-tagged open reading frames (ORFs) from the human ORFeome, a project designed to clone ORFs from the human genome into expression vectors for protein purification. Subsequently, complexes with these proteins were affinity-purified and analyzed by mass spectrometry. The resulting data revealed more than 50,000 interactions, and network analysis indicated 354 “communities” of protein complexes, generating a collective network that the researchers named BioPlex (for Biophysical Interactions of ORFeome-Based Complexes). This network analysis was further successful in assigning characteristics of proteins, such as localization and function, by position within the network. Most importantly, and satisfyingly, proteins with similar domains were clustered together in the network, implying a role for structure in the formation of protein-protein interactions. These findings suggest a basis for new bioinformatics approaches enabling the inference of protein function, localization, and structure through the analysis of interacting partners, or vice versa. Furthermore, the examination by these methods of sub-networks from individuals may shed light on signaling circuits that are important for specific diseases. Huttlin *et al*. provide an elegant example of such analyses by examination of the B- and A-isoform sub-network of the VAMP-associated proteins (VAPs): They report that interactions of the mutated form of VAPB with some of its targets were different from those observed with the wild-type protein.

Hein *et al*. ^43^ employed a different approach to analyze the human interactome, using proteomics applied in three quantitative “dimensions”: interactions between proteins, the stoichiometry of interacting proteins, and their cellular abundance. The researchers generated a bacterial artificial chromosome library with GFP-tagged proteins and expressed the library in a HeLa cell line. Subsequently, interacting complexes were immunoprecipitated by using an anti-GFP antibody, and protein quantities were determined through a single run of liquid chromatography-tandem mass spectrometry. These data revealed both known complexes and interactions as well as previously unknown complexes. Interestingly, the previously unannotated complexes identified in this work were typically composed of fewer proteins than the known complexes. Further validation of these results indicated an interaction between the selected low-abundance protein KIAA1430 and the anaphase-promoting complex/cyclosome (APC/C), suggesting that this method is indeed capable of detecting transient interactions exceeding the limits detectable through traditionally available methods. Moreover, the stability of the interaction was correlated with interaction stoichiometry. The authors concluded that although strongly associated complexes can be identified without stoichiometric analysis, weaker interactions between transiently interacting sub-stoichiometric proteins may be missed, potentially obscuring important linkages between complexes that collectively yield a global representation of network connectivity in cells.

It is useful to note that while both the BioPlex and three-dimensional quantitative interactome studies discussed above yield a coverage far exceeding that which has been obtained from traditional interactome studies, neither work can claim to be comprehensive. In fact, researchers are currently expanding the BioPlex network through additional rounds of AP-MS analysis to address this point. It would be interesting to determine the degree to which the resulting networks overlap in the two studies.

Recently, Savitski *et al*. developed a clever approach to identify chemical-protein interactions (for example, the interactions between a drug and its protein target) by using mass spectrometry ^44^. The researchers implemented a cellular thermal shift assay to identify proteins for which ligand binding affects thermal stability. By this approach, cells, cultured with and without drug, are heated to a range of temperatures, thereby inducing protein denaturation. At each temperature, soluble proteins are extracted and identified by mass spectrometry. Protein thermal stability profiles affected by drug treatment thus can be determined, suggesting putative drug targets. Adding to their other results, researchers used this method to identify more than 50 targets of the kinase inhibitor staurosporine.

**Figure 2.**
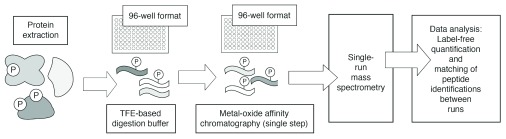
The EasyPhos pipeline for quantitative and dynamic phosphoproteomics. Basic steps in the EasyPhos protocol are indicated. Steps that can be performed in a 96-well format are indicated as such. Phosphorylation sites are indicated graphically with a circled “P”; peptides are indicated as wavy lines. Collectively, the EasyPhos protocol is more streamlined than commonly used alternatives. The EasyPhos method uses a tetrafluoroethylene (TFE)-based digestion buffer, in place of a more labor-intensive peptide desalting step, prior to phosphopeptide enrichment; this further facilitates the implementation of EasyPhos in a 96-well format. The metal-oxide affinity chromatography techniques implemented typically using microcolumns are popular and established means by which phosphopeptides may be enriched or separated from non-phosphorylated peptides for subsequent mass spectrometry.

In a study that complements the ones above, Humphrey *et al*. in Matthias Mann’s laboratory recently developed a method they refer to as EasyPhos ^45^. The EasyPhos method enables the rapid quantification of protein phosphorylation on a proteome-wide scale *in vivo* ( Figure 2). EasyPhos indeed offers many improvements over traditional mass spectrometry-based proteomic tools. In particular, a smaller amount of starting sample is required as compared with amounts required for conventional methods. In EasyPhos, the sample is digested by using tetrafluoroethylene instead of urea or methods for filter-assisted sample preparation. Hence, desalting and lyophilizing the sample become unnecessary, and a single parallel enrichment of phosphopeptides becomes possible before liquid chromatography-tandem mass spectrometry analysis. The ease and speed with which EasyPhos can be used make it suitable for the analysis of dynamic phosphoproteomes and provide a convenient alternative to widely applied approaches based on SILAC (stable isotope labeling of amino acids in cell culture). For example, Humphrey *et al*. used EasyPhos to analyze dynamic insulin signaling in the mouse liver. From this work, many unknown phosphorylation events were detected and mapped to a time-resolved atlas. Through this atlas, the researchers revealed a link between insulin signaling and lipid storage in the liver as well as an interconnection between insulin and fibroblast growth factor signaling. As EasyPhos enables detection of the timing of phosphorylation events, Humphrey *et al*. were able to detect the directionality of signal transduction. Rather surprisingly, at least for the Akt signaling response analyzed in this work, signal was not transduced linearly from the cell surface to the nucleus. By incorporating a temporal dimension into the analysis of cell signaling, EasyPhos and other methods yet to be invented, coupled with improved sample preparation, labeling techniques, and enrichment, promise proteomic studies that will improve our understanding of signaling circuits toward the development of more accurate computational signal response simulations.

## The integration of pathway analyses at the single-cell level

Although genetic screens and proteomic profiling studies provide important information regarding the makeup of pathways as well as mechanistic insight at the protein level, the most important advancement in dissecting signaling circuitry may result from the integration of many such approaches. Furthermore, the application of such integrative approaches at the single-cell level is a particularly exciting development, since in many instances data averaged over a population of cells may obscure important signaling mechanisms. For example, important protein interactions may occur in only a subset of cells ^46^, necessitating analysis at the single-cell level for detection. Below, we present several recent examples of studies at the single-cell level coupling microscopy or transcriptional analysis with sophisticated computational methods.

**Figure 3.**
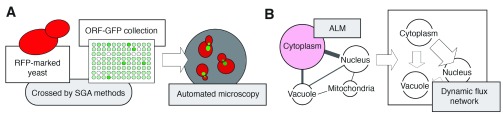
Overview of the generation of abundance localization maps (ALMs) and a dynamic flux network from the analysis of protein localization and abundance in yeast. ( **A**) A collection of yeast strains containing gene-green fluorescent protein (GFP) fusions were crossed with a yeast strain containing a cytosolic red fluorescent protein (RFP) chimera by synthetic genetic array (SGA) methods. SGA methods were developed as a means to cross a haploid yeast strain containing a mutant allele with another yeast mutant strain of opposite mating type for the subsequent selection of a haploid double mutant. By this approach, yeast strains were generated where the cytoplasm of the yeast cells can be visualized in red, and the localization of each target protein can be observed as green fluorescence. Subsequently, the yeast strains are imaged by automated microscopy. ( **B**) The acquired data are integrated into ALMs. Changes in these maps in response to cell stress, chemical treatments, and genetic perturbations can be captured in dynamic flux networks, as presented. Abbreviations: ORF, open reading frame.

Chong *et al*. ^47^ present an exceptional study from the laboratories of Brenda Andrews, Charlie Boone, Jason Moffat, and Andrew Moses quantitatively analyzing protein localization and abundance on a proteome-wide scale in *S. cerevisiae*. This work also assesses dynamic changes in the proteome in response to chemical treatments and genetic mutation. For these analyses, the researchers used high-throughput genetics, in the form of synthetic genetic array methodologies developed in the Boone and Andrews laboratories ^48, 49^, to cross a large collection of yeast strains expressing ORF-GFP fusions with a cytosolic red fluorescent protein (RFP) marker, thereby delineating cell boundaries in red ( Figure 3A). With this collection of yeast strains for microscopy, the authors implemented a machine learning approach to train software for the recognition of 16 subcellular compartments using a subset of proteins that each localize to only one compartment. The full collection of ORF-GFP strains with RFP were visualized by automated microscopy, and analysis of the resulting images enabled assignment of proteins to the predefined subcellular compartments. Both the localization and abundance of proteins were determined in this manner. From these data, an abundance localization map (ALM) was generated, with cell compartments as hubs and proteins as nodes within the network. A large number of proteins localized to more than one compartment, linking one hub to another. The researchers tracked changes in protein localization and abundance in response to chemical treatment with rapamycin and hydroxyurea, mimicking nutrient deprivation and DNA replication stress, respectively. The yeast collection was also assessed for changes in protein dynamics upon deletion of *RPD3*, a gene encoding a lysine deacetylase. An ALM was generated for each of the conditions tested, and changes in the ALMs from one condition to the next were quantified, yielding a flux network indicating the dynamic nature of the proteome ( Figure 3B). Interestingly, the flux network revealed changes for a given protein in either localization or abundance, but seldom both in response to environmental or genetic perturbation. Collectively, this work provides a framework for future applications of high-throughput microscopy while exemplifying the utility in integrative approaches for the analysis of protein dynamics.

Wachsmuth *et al*. also investigated the dynamic proteome through techniques integrating high-throughput microscopy with automated image analysis ^50^. These researchers developed methods for high-throughput fluorescence correlation spectroscopy (HT-FCS), which in conjunction with confocal microscopy allowed them to assess the localization of fluorescently labeled single molecules in living human cells. Through automated image acquisition followed by automated FCS, researchers were able to capture many images from one sample, enabling high-throughput analysis. By this approach, the dynamics of 53 nuclear proteins were analyzed in HeLa cells, and three clusters of proteins were identified, indicating chromatin binders, single non-chromatin binders, and complex non-chromatin binding proteins. The authors were also able to track a single cell throughout its cell cycle by time-lapse imaging. In this study, the researchers presented software for the analysis of fluctuations between time points separated by as little as 10 minutes. This method was used to successfully track chromosomal passenger complex assembly throughout the cell cycle. Through better labeling and improved time resolution, HT-FCS carries the potential to complement mass spectrometry as an approach for determining the dynamic proteome *in vivo*.

Exemplifying the utility in single-cell studies, Gary Nolan and Dana Pe’er and colleagues have advanced the analysis of single-cell mass cytometry data to more fully define signaling responses. Krishnaswamy *et al*. ^51^ developed and applied computational methods to analyze mass cytometry data quantifying the abundance of 20 protein epitopes at a range of time points following two different types of T-cell receptor activation in B6 mice. Levine *et al*. ^52^ used mass cytometry to profile surface and intracellular signaling proteins in healthy cells and in cells exhibiting acute myeloid leukemia. In this work, the authors determined a gene expression signature predictive of cell survival while validating new methodologies for the analysis of large single-cell data sets from a heterogeneous cell population.

Although the rate at which single-cell studies have been undertaken has accelerated significantly over the past few years, studies of gene expression in single cells date back to at least 1992 ^53– 55^. Recently, these gene expression data have been used to dissect cell signaling pathways. An elegant example of such a study is presented by Moignard *et al*. ^56^, who used a combination of single-cell gene expression data and computational modeling to analyze signaling events that regulate early blood development. This experimental design makes use of the fact that early progenitors of blood cells express Flk1 but that at very late stages, Flk1 expression may be lost, with hematopoietic potential evident in cells expressing Runx1. With a Runx1-Ires-GFP mouse model, precursor blood cells were sorted into five groups in four stages according to the expression status of Flk1 and GFP and time of harvesting. In this study, the expression profiles of 33 transcription factors, nine markers, and four housekeeping genes were determined for 3,934 cells. The cells were clustered into three groups by unsupervised hierarchical clustering according to the respective gene expression profiles, regardless of whether blood-associated genes were expressed in a given cell. Interestingly, cells from different stages of development could be found at each cluster, suggesting that cells do not commit at a uniform point in time. Single-cell analysis is thus important in determining the exact time at which this commitment occurs. To analyze single-cell data, the researchers constructed a diffusion map, placing each of the cells on a three-dimensional map, and the distance between cells was determined by their degree of similarity. This map eventually resembled the relevant developmental stages, suggesting that this approach can be used to order genes and proteins during development. Moignard *et al*. used these data to generate predictive models, which they subsequently validated. Although these computational models neglect the fact that a given gene may be upregulated or downregulated rather than being transcriptionally on or off, this study does provide a first-of-its-kind framework for deciphering signaling networks important in mammalian organogenesis from single-cell expression data without any prior knowledge.

## Areas of future development

Considered collectively, the approaches discussed here present advancements toward mapping signaling pathways through genetic, proteomic, and integrative methods, particularly in identifying signaling responses in individual cells within a metazoan organism or cell population. It is important to note, though, that these larger-scale studies can be very informatively complemented with more focused work on key genes and proteins. The degree to which apparently diverse approaches are in fact integrated within these studies will likely expand in the coming years. In general, published work already involves a combination of genetic, molecular, biochemical, and cell biological approaches, and bioinformatics further contributes to the rich and new data sets that are being presented. In order to effectively define signaling circuitry, this integration is critical, in conjunction with the continued development of new technologies for the granular analysis of protein function. Although it is clear that we have yet to fully characterize even a single signaling pathway, the future holds exceptional promise toward identifying new signaling mechanisms and, consequently, a clearer understanding of eukaryotic cell biology. 

## Abbreviations

ALM, abundance localization map; AP-MS, affinity purification mass spectrometry; CRISPR/Cas, clustered regularly interspaced short palindromic repeats/CRISPR-associated; FCS, fluorescence correlation spectroscopy; GFP, green fluorescent protein; HT-FCS, high-throughput fluorescence correlation spectroscopy; LPS, lipopolysaccharide; ORF, open reading frame; RFP, red fluorescent protein; sgRNA, single-guide RNA; TNF, tumor necrosis factor. 
